# Notes of Clinical Lectures in Atlanta Medical College

**Published:** 1866-04

**Authors:** A. G. Thomas


*Notes of Clinical Lectures in Atlanta Medical College.*—By A. G. THOMAS, M. D.

Of the sixty-five cases presented to the class of Atlanta Medical College, from the middle of November, 1865, to the first of January, 1866, I propose to present to the readers of the Atlanta Medical and Surgical Journal, some brief notes of the most interesting ones which it was my good fortune to have the opportunity of taking from the lectures as delivered. I have the notes of only a few lectures now arranged for the press, but will furnish others in a series of articles for succeeding numbers of the Journal.

NOVEMBER 21.

*Dr. J. G. Westmoreland.*—A case of Icterus—Jaundice. The usual yellow hue observable upon the skin and albuginea of the eye, is seen in this case. In regard to the pathology, he said the profession did not agree. The majority, perhaps, favor the opinion that the secernent function of the liver is deranged, and from that disturbance the principles in the blood, from which bile is manufactured in the liver, being retained in the circulation, are deposited in the tissues, which are found tinged with yellow. In accordance with this theory, the treatment usually adopted is such as is applicable to inactivity of the liver, cholagogue and tonic remedies. This is the rational treatment for such pathological condition, and would be found generally useful, were this the true pathology of the disease. The symptoms, however, in this case, as in all others, cannot be satisfactorily explained upon this theory, in my judgment.

In the first place, bile does not exist, as such, in the blood; and is not found deposited, as in jaundice, when from cirrhosis or other diseases of the liver, the secretion of bile is interfered with. On the other hand, where post mortem examination has revealed the presence of biliary calculi in the bile duct, obstructing the passage of bile from the liver to the bowel, the prominent symptom of jaundice, yellow hue, was found to exist. These facts seem to prove that bile is secreted by the liver before it is deposited in the skin and eyes, and that it is thus disseminated by being taken into the circulation through absorption, when obstruction exists to its passage into the bowel. In ordinary jaundice the obstruction usually exists in the duodenum, at the mouth of the bile duct. The rational treatment, according to this theory, would be to relieve that condition of the bowel, upon which the obstruction depends. Duodenitis, or engorgement and irritability of the dodenum, is the condition to which we should direct our remedies; and the rational course of procedure for the relief of this local derangement, is, according to my observation, the most effectual treatment of jaundice. Blisters over the epigastrium, mucilaginous drinks, opiates and alkalies, usually fill the indication.

A case of Intermittent Fever was also exhibited, the pathology of which he alluded to as a diseased or poisoned condition of the medulla spinallis, by that subtle, unknown agent called malaria. Intermittent and Remittent fever, he considered identical, and consequently required the same treatment. The great antiperiodic and febrifuge, quinine, generally removes the abnormal condition of this nervous centre, and in the restoration of its functions, the functional disturbance of other organs is abated. He does not usually premise the treatment by the use of colagogues, cathartics, emetics, &c., but advises quinine at the first abatement of fever.

*A case of Rheumatism in a scrofulous constitutian.*—There exists constitutional predisposition to, or peculiarity of conformation suited to certain diseases, which is called *diathesis*. The scrofulous diathesis subjects the individual, from depressing influences, to the development of the degenerate substances, scrofulous and turberculous deposits. The want of nourishing diet, exposure to damp and cold atmosphere, and an attack of ordinary disease, such as the case of rheumatism before you, may depress the energies of the system to the point at which these degenerate accumulations are produced; and in the case before you there is a striking example of the difficulty met with in treating common diseases in this form of constitution. The scrofulous deposits have doubtless occurred upon the very tissues involved in rheumatism, and may protract this disease, which of itself might be speedily cured, to an indefinite period. The skin, the bones—particularly about the joints—and the lymphatic glands, (especially the cervical,) are the tissues most subject to scrofulous deposits. The mucous surfaces (particularly of the air passages and bowels,) the lungs and brain, are the parts in which tubercular deposits are likely to occur.

In the treatment of rheumatism, acute articular rheumatism, unconnected with constitutional contamination, the course to be pursued is simple and effectual. Like intermittent and remittent fever, the primary disturbance most probably exists in the medulla spinalis, and of a character suited to the favorable action of that indispensable nervous tonic, quinine. The migratory character of the disease, often leaving the part affected, and suddenly attacking a healthy joint, without the usual results of ordinary inflammation, establishes the fact that rheumatism is not a local disease, and that remedies directed to the painful and swollen joint cannot be permanently curative. Blisters to the spine, together with quinine and opium, arrest the progress of acute articular rheumatism, with as much certainty as the paroxysms of intermittent fever are broken up. The following is a favorite combination:

℞ Quiniæ Sulph. grs. xx.

Pulv. Doveri grs. xxx.

M. et ft. chat No. iv.

Give one, morning, noon and night.

Or the following:

℞ Quinæ grs. xx.
Pulv. Opii grs. vjii.
Ft. Pillulæ no. vjii.
Sig. Two to be given night and morning.

